# Best of general and upper GI surgery in 2023

**DOI:** 10.1093/bjsopen/zrae002

**Published:** 2024-01-24

**Authors:** Marcel André Schneider

**Affiliations:** Associate Editor (Upper GI, SoMe)

The year 2023 brought a wealth of interesting studies in the realm of general and upper GI surgery to *BJS Open*, and it was a delight to see them in print and gaining recognition. All of these papers are the fruits of the authors’ year-long labour and we are grateful to them for choosing *BJS Open* as the media to publish their meaningful results. Here, I would like to highlight several papers that have extended my horizon during the last year.

Our comprehensive reviews are a great way to gain in-depth knowledge of surgical topics. Such an educational summary was the review on fluorescence-guided surgery, an innovative technique that can be utilized for various surgical purposes. The article by Sutton *et al*. delves into the four main applications for which it can be employed, namely tissue perfusion, lymph node assessment, better anatomical depiction of vital structures and tumour tissue imaging^1^. This is a fascinating paper that explains the current and future applications of this technique and is a must-read for every surgeon.

With ChatGPT and other large language models (LLMs) emerging as significant artificial intelligence breakthroughs in 2023, the *BJS Open* leading article on the utilization of LLMs in surgical science by Janssen *et al*. gained immense popularity, with over 5000 views and 1000 downloads so far^2^. The corresponding tweet by the last author, Marc Besselink, has been viewed over 220 000 times. The article shows in a concise manner how ChatGPT can be employed to produce and enhance research-related text such as grant proposals, protocols or study outlines. It also outlines potential future applications such as data extraction, code generation or aiding in clinical decision-making. Whether we like it or not, LLMs are here to stay and will profoundly impact the entire academic publishing and editing process, with the full impact likely only becoming apparent in a few years.

Another paper that particularly spiked my interest was the well-conducted meta-analysis by Frassini *et al*. on prophylactic mesh placement following elective and emergency surgery^3^. Incisional hernia remains a significant postoperative concern, and the analysis clearly confirmed that midline laparotomy closure with prophylactic mesh augmentation significantly reduces incisional hernia development. With such compelling evidence available, it makes me wonder why most surgeons (including myself) still don't perform it routinely?

Another highly relevant study was the systematic review by Ashmore *et al*. on the identification of malnutrition in emergency surgical patients^4^. They discovered an array of seven malnutrition screening and nine assessment tools, with BMI and albumin being the most common. While we encounter these patients on a daily basis, this study made me realize how little we understand and address malnutrition. In a population of emergency surgical patients with increasing co-morbidities and advancing age, a standardized assessment of nutritional status and initiation of appropriate interventions are essential.

Last but not least, the short report by Wang *et al*. describing the utilization of a novel device that evaluates gastric electrophysiology and function at high resolution in patients who have undergone oesophagectomy and experience delayed gastric conduit emptying intrigued me^5^. This device offers a novel diagnostic option for patients with functional upper GI problems, and so far, reliable options for conduit function assessment are lacking. It will be interesting to observe if this device, which is currently undergoing evaluation in several trials, can potentially make a difference for patients with functional gastric disorders.
